# Vertebral osteomyelitis in patients with infective endocarditis: prevalence, risk factors and mortality

**DOI:** 10.1007/s10096-025-05041-8

**Published:** 2025-01-21

**Authors:** S. Douiyeb, K. C. E. Sigaloff, E. G. Ulas, M. G. J. Duffels, O. Drexhage, T. Germans, J.F.P. Wagenaar, D. T. P. Buis, T. W. van der Vaart, C. H. van Werkhoven, J. M. Prins, V. A. W. M. Umans

**Affiliations:** 1 Department of Internal Medicine, Amsterdam UMC location AMC, Amsterdam, The Netherlands; 2 Amsterdam Institute for Infection and Immunity, Amsterdam, The Netherlands; 3 Department of Internal Medicine, Amsterdam UMC location Vrije Universiteit Amsterdam, Amsterdam, The Netherlands; 4 Department of Cardiology, Noordwest Hospital, Alkmaar, The Netherlands; 5 Department of Internal Medicine, Noordwest Hospital, Alkmaar, The Netherlands; 6 Julius Center for Health Sciences and Primary Care, UMC Utrecht, Utrecht University, Utrecht, The Netherlands

**Keywords:** Infectious endocarditis, Vertebral osteomyelitis, Spondylodiscitis, Age, Enterococci

## Abstract

**Purpose:**

Infective endocarditis (IE) can be complicated by vertebral osteomyelitis (VO). This study investigates risk factors associated with VO in patients with infective endocarditis, and 6-month mortality and relapse rates in patients with IE and concomitant VO.

**Methods:**

We performed a observational study in two hospitals between September 2016 and October 2022. Patients with possible or definite IE according European Society of Cardiology (2015) modified criteria were retrieved from the local endocarditis team registries. The VO diagnosis was based on radiological signs, irrespective of clinical symptoms. Multivariable logistic regression analysis was performed to identify risk factors for vertebral osteomyelitis.

**Results:**

We included 633 consecutive patients with IE. A total of 229 (36.2%) patients had prosthetic valves and 127 (20.1%) had cardiac implantable electronic devices. The most frequent causative micro-organism was *Streptococcus species* (217, 34.3%), followed by *Staphylococcus aureus* (167, 26.4%). VO was diagnosed in 73 patients (11.5%, 95% CI 9.0%-14.0%). *Enterococcus spp.*(OR 2.48, 95% CI 1.31–4.52) and age (OR 1.04 per year, 95% CI 1.02–1.06) were independently associated with concomitant VO. The 6-month mortality risk did not differ between patients with (16/73, 21.9%) or without (110/560, 19.6%) VO (HR 1.13, 95% CI 0.67–1.91). Relapse rate was higher in patients with VO but the difference was not statistically significant (16.1 vs. 7.5%, OR 3.62, 95% CI 0.94–13.34).

**Conclusions:**

Twelve percent of patients with IE also had VO. Among older patients and patients with IE caused by enterococci, there should be a higher index of suspicion for vertebral infection.

## Introduction

The association between infective endocarditis (IE) and vertebral osteomyelitis (VO) was first described in 1965 [1]. The pathogenesis is assumed to be hematogenous spread of the bacteria from the heart to the highly vascular bone marrow of the vertebral bodies. The prevalence of VO in patients with IE can range from 8–19% [2–5]. While the prognosis appears to be comparable for patients with and without VO, studies show that additional interventions, such as vertebral surgery and abscess drainage, may be indicated in some patients with VO in order to achieve infection control [4, 6]. Furthermore, a concomitant vertebral osteomyelitis sometimes requires prolongation of antibiotic treatment [6].

Determining which patients with infective endocarditis require further diagnostics and treatment for vertebral osteomyelitis presents a clinical challenge. Several risk factors have been associated with VO in patients with IE, including male gender, old age and infections caused by enterococci or streptococci [2, 3, 7]. However, the epidemiological profile of IE may vary between geographic regions and over time. Changing demography, comorbidity, healthcare usage and the increasing implantation of prosthetic material all contribute to changing epidemiology of IE and may alter the relationship between IE and VO [8]. In addition, studies conducted on the association between IE and VO are often retrospective, single centre or tertiary-care based, which may reduce the external validity of epidemiological and prognostic findings [2–4, 7].

The primary objective of this study was to identify risk factors associated with VO in a cohort of patients with infective endocarditis evaluated during the past decade in a university and a general teaching hospital. Secondary objectives included quantification of 6-month mortality and relapse rates in IE patients with and without VO.

## Materials and methods

### Study design

A prospective observational study was carried out at two hospitals in The Netherlands: the Noordwest Hospital (NWZ), a large teaching hospital and the Amsterdam UMC, a university hospital with a cardiothoracic surgery unit. In both hospitals, all patients with suspected endocarditis are discussed in the local Endocarditis team. This team consists of a cardiologist, infectious disease specialist, medical microbiologist, nuclear radiologist and, in Amsterdam UMC, a cardiothoracic surgeon. Meeting notes and treatment plans are registered in the Electronic Medical Record (EMR). All data for the present study was prospectively collected from September 2016 until October 2022. The study was approved by the local ethics committees of Amsterdam UMC and NWZ. In the Amsterdam UMC, patients were given the opportunity to object via an opt-out procedure. In the NWZ informed consent was waived, as the study was considered standard evaluation of quality of care.

### Study population

All consecutive patients aged 18 years and older diagnosed with definite or possible infective endocarditis according to the ESC guidelines 2015 were identified and discussed in the endocarditis team [9]. If the diagnosis of endocarditis was rejected by the endocarditis team, patients were excluded. If patients were discussed in the Endocarditis teams of both the NWZ hospital and Amsterdam UMC, the patient was included only once.

### Data collection

Demographic variables, comorbidities, presence of prosthetic material (any cardiac prosthetic material such as heart valves or cardiac implantable electronic devices (CIED)), cardiac involvement, causative micro-organism, complications of IE and treatment were collected from the EMR. The diagnostic work-up of suspected or proven IE was according to the European Society of Cardiology (ESC) guidelines [9]. In the Amsterdam UMC additional information about the modified criteria for the diagnosis of IE according to the ESC guidelines was collected [9]. Embolic events were classified as cerebral, visceral, osteoarticular (other than VO) or pulmonary. Visceral embolic events included emboli in liver, kidney or spleen.

The diagnosis of VO was based on radiological signs of VO, irrespective of clinical symptoms [6]. Imaging was performed when VO was clinically suspected or was performed as part of the diagnostic work-up of IE. Imaging included a CT-, MRI- and/or ^18^F-FDG-PET/CT-scan, performed at the treating physicians discretion and without involvement of the study team. Diagnostic criteria for VO consisted of signs of paravertebral, epidural or facet inflammation, edema of the vertebrae or disc, or abscess formation on either MRI or CT-scan. ^18^F-FDG-PET/CT was considered positive for VO if FDG-uptake was increased in osseous structures, intervertebral disc space and/or endplate with surrounding soft tissue [2, 10].

All patients received an empirical, and if possible, a targeted 4 to 6-week antimicrobial treatment for infective endocarditis according to the Dutch national guidelines for treatment of infective endocarditis [11]. The duration of intravenous treatment was 6 weeks in case of VO. Relapse was defined as recurrent episode of IE with the same causative micro-organism as the primary episode. Patients treated in the NWZ were prospectively followed for 6 months and, therefore, data on relapse and 6-month mortality was available for all patients. Data on relapse was not available for the Amsterdam UMC. Information on 6-month mortality of the patients treated in the Amsterdam UMC was collected from the electronic health record and if not available the municipal death records.

### Primary and secondary outcome measures

The primary outcome measure were the risk factors associated with the presence of VO in patients with IE. Secondary outcome measures were 6-month mortality and relapse rates.

### Statistical analysis

Categorical data were described as numbers and percentages, continuous data using the appropriate descriptive statistics for normally and non-normally distributed variables. Differences between groups were analysed with Chi-Square-test for categorical data and unpaired T-Tests for normally distributed continuous data; if data was non-normally distributed a non-parametric test was used, such as the Mann-Whitney-U-test. Univariable analysis was performed using logistic regression to identify variables associated with VO. Candidate variables were selected based on previous studies and on biological plausibility, and included age, sex, the presence of cardiac device or prosthetic valve, diabetes mellitus and the type of causative micro-organisms [2, 3, 7]. Given the negligible amount of missing data, no imputation methods were applied. Two additional sensitivity analyses were performed to identify risk factors in patients without known VO at the time of IE diagnosis and in patients with a definite IE. For the second sensitivity analysis, data were only available for patients in the Amsterdam UMC cohort. Variables associated with p-value < 0.1 were subsequently analyzed in multivariable logistics regression models. Kaplan-Meier curves were used to estimate the survival probability over the 6-month follow-up and groups were compared using Cox regression. Relapse rates were compared using logistic regression. All analyses were performed using R studio 4.2.1 (R Core Team 2022) and a p-value below 0.05 was considered statistically significant.

## Results

### Patient characteristics

From September 2016 through October 2022, 633 consecutive patients with endocarditis were included, 165 patients from the NWZ and 468 from Amsterdam UMC. All were treated for IE by recommendation of the endocarditis team. An overview of the baseline characteristics is given in Table 1. The majority was male (71.6%), with a median age of 69 years (IQR 58–77). A total of 36.2% and 20.1% of all patients had a prosthetic valve or CIED in situ, respectively. The aortic valve was most frequently affected (51.2%), followed by the mitral valve (19.9%) and CIED-related infections (11.3%). The most frequent causative micro-organism was *Streptococcus* species (34.3%) followed by *Staphylococcus aureus* (26.4%). A total of 28% of patients experienced systemic embolization, respectively cerebral (13.3%), visceral (7.9%), osteoarticular (other than VO) (6.2%) and pulmonary emboli (5.7%). ^18^F-FDG-PET/CT was performed in 374 patients (59.1%).

**Table 1:** Baseline characteristics of patients with infective endocarditis with or without concomitant vertebral osteomyelitis

	With vertebral osteomyelitis (*n* = 73)	Without vertebral osteomyelitis (*n* = 560)	*p*-value
Demographics			
Sex, male	55 (75.3)	398 (71.1)	0.5
Age (median, IQR)	73 (66–80)	68 (57–76)	*p* < 0.001
Medical history			
History of IE	6 (8.2)	50 (8.9)	1
Diabetes mellitus*	16 (21.9)	106 (18.9)	0.62
Prosthetic material			
Prosthetic valve	19 (26.0)	210 (37.5)	0.08
CIED	15 (20.4)	112 (20.0)	1
Cardiac involvement			
Aortic valve	36 (49.3)	288 (51.4)	0.89
Mitral valve	20 (27.4)	106 (18.9)	0.11
Pulmonary valve	0 (0.0)	8 (1.4)	0.76
Tricuspid valve	2 (2.7)	24 (4.3)	0.58
CIED	6 (8.2)	66 (11.8)	0.81
No cardiac signs of IE	9 (12.3)	68 (12.1)	0.54
Presence of vegetation	368 (65.7%)	55 (75.3%)	
Causative micro-organism			
*Streptococcus* spp.	25 (34.2)	192 (34.3)	1
*Staphylococcus* aureus	22 (30.1)	145 (25.9)	0.53
Enterococci	17 (23.3)	55 (9.8)	*p* < 0.01
CNS	7 (9.6)	41 (7.3)	0.65
HACEK-group	-	18 (3.2)	0.24
Culture negative	-	53 (9.5)	0.01
Other	2 (2.7%)	56 (10.0)	0.07
Embolic events			
Cerebral	10 (13.7)	74 (13.2)	1
Visceral	8 (11.0)	42 (7.5)	0.42
Osteoarticular (other than VO)	10 (13.7)	28 (5.2)	0.007
Pulmonary	5 (6.8)	31 (5.5)	0.85
PET-CT-scan performed	58 (79.5)	316 (56.4)	*p* < 0.01

Data are reported as number of patients (%), unless otherwise specified

CIED, Cardiac implantable electronic devices; CNS, coagulase negative Staphylococci; IE, infectious endocarditis; SD, standard deviation; VO, vertebral osteomyelitis

*The proportion of missing data for the variable diabetes mellitus was 0.3% (2/633)

### Concomitant vertebral osteomyelitis

A total of 73/633 patients (11.5%, 95% CI 9.0%-14.0%) with IE had concomitant vertebral osteomyelitis. VO was more frequently diagnosed in patients who underwent ^18^F-FDG-PET/CT-scan (15.5% vs. 5.8; *p* < 0.01). The diagnosis VO was confirmed by CT (2 case), MRI (14 cases), 18F-FDG-PET/CT (37 cases) and MRI and ^18^F-FDG-PET/CT combined (20 cases). In 51% (37/73) of VO patients, VO was diagnosed before infective endocarditis.

Patients with VO were older (median age 73 vs. 68 years, *p* < 0.001) compared to patients without VO. Prosthetic cardiac valves were less frequently present in patients with VO, but this difference did not reach statistical significance (26% vs. 37.5%; *p* = 0.08). Other demographics, such as a history of infective endocarditis, comorbidities, and affected valves were comparable between both groups (Table 1) There were relatively more cases of IE by enterococci in the patients with concomitant VO (23.3% vs. 9.8%; *p* < 0.01). Cerebral, visceral, osteoarticular or pulmonary embolic events occurred more frequently in patients with concomitant VO (42.5% vs. 26.8% (*p* < 0.01).

Ten percent of patients with VO had a previous history of vertebral osteomyelitis and 77% complained of backpain. VO was located at the lumbosacral, thoracic, cervical and multiple levels in 35 (47.9%), 15 (20.5%), 8 (11.0%) and 15 (20.5%) cases, respectively. Spinal abscess, vertebral collapse and neurological impairment due to VO occurred respectively in 16 (21.9%), 6 (8.2%) and 4 cases (5.5%). Five (6.8%) patients required vertebral surgery. Abscess drainage was performed in one case.

### Primary outcome

In univariable analyses, age (OR 1.04, 95% CI 1.02–1.06) and enterococci (OR 2.79, 95% CI 1.48–5.05) were associated with VO. In multivariable analyses, the same factors were statistically significant (Table 2.).

**Table 2:** Uni- and multivariable logistic regression analysis of 6-month risk of vertebral osteomyelitis in patients with endocarditis

Risk factor	Univariable analysis OR (95% CI)	Multivariable analysis OR (95% CI)	Sensitivity analysis IE before VO OR (95% CI)****	Sensitivity analysis Duke definite OR (95% CI)****
Age	1.04 (1.02–1.06)***	1.04 (1.02–1.06)***	1.04 (1.00-1.07)*	1.04 (1.00-1.07)*
Sex	1.25 (0.72–2.24)	-	-	-
CIED at admission	1.04 (0.55–1.85)	-	-	-
Diabetes mellitus	1.22 (0.65–2.16)	-	-	-
Prosthetic valve at admission	0.59 (0.33-1.00)	-	-	-
*Streptococcus spp.*	1.00 (0.59–1.65)	-	-	-
*Staphylococcus aureus*	1.23 (0.71–2.08)	-	-	-
*Enterococcus spp.*	2.79 (1.48–5.05) ***	2.48 (1.31–4.52)**	3.64 (1.63–7.68)***	2.79 (1.06–6.79)*
Coagulase negative *Staphylococci*	1.34 (0.53–2.94)	-	-	-

*p < 0.05; **p < 0.01; ***p < 0.001

**** Excluding those patients in whom vertebral osteomyelitis was already diagnosed at the moment of infective endocarditis diagnosis. CIED, cardiac implantable electronic device

### Sensitivity analyses

In both sensitivity analyses, the same risk factors were associated with VO. Of the 596 patients without a previously established diagnosis of VO at the time of IE diagnosis, 36 patients were later diagnosed with VO (6.0%). In univariable analyses age and enterococci were associated with a higher risk of VO (Table 2). In multivariable analyses, age (OR 1.04, 95% CI 1.00-1.07) and enterococci (OR 3.64, 95% CI 1.63–7.68) remained associated with the presence of VO. In patients with a definite endocarditis (*n* = 314/469 from the Amsterdam UMC) the prevalence of VO in this population was 33/314 (10.5%), which was comparable to the prevalence found in the main analysis. Age (OR 1.03, 95% CI 1.00-1.07) and enterococci (OR 2.79, 95% CI 1.06–6.79) also remained associated with the presence of VO.

### Secondary outcomes

**Fig. 1:**
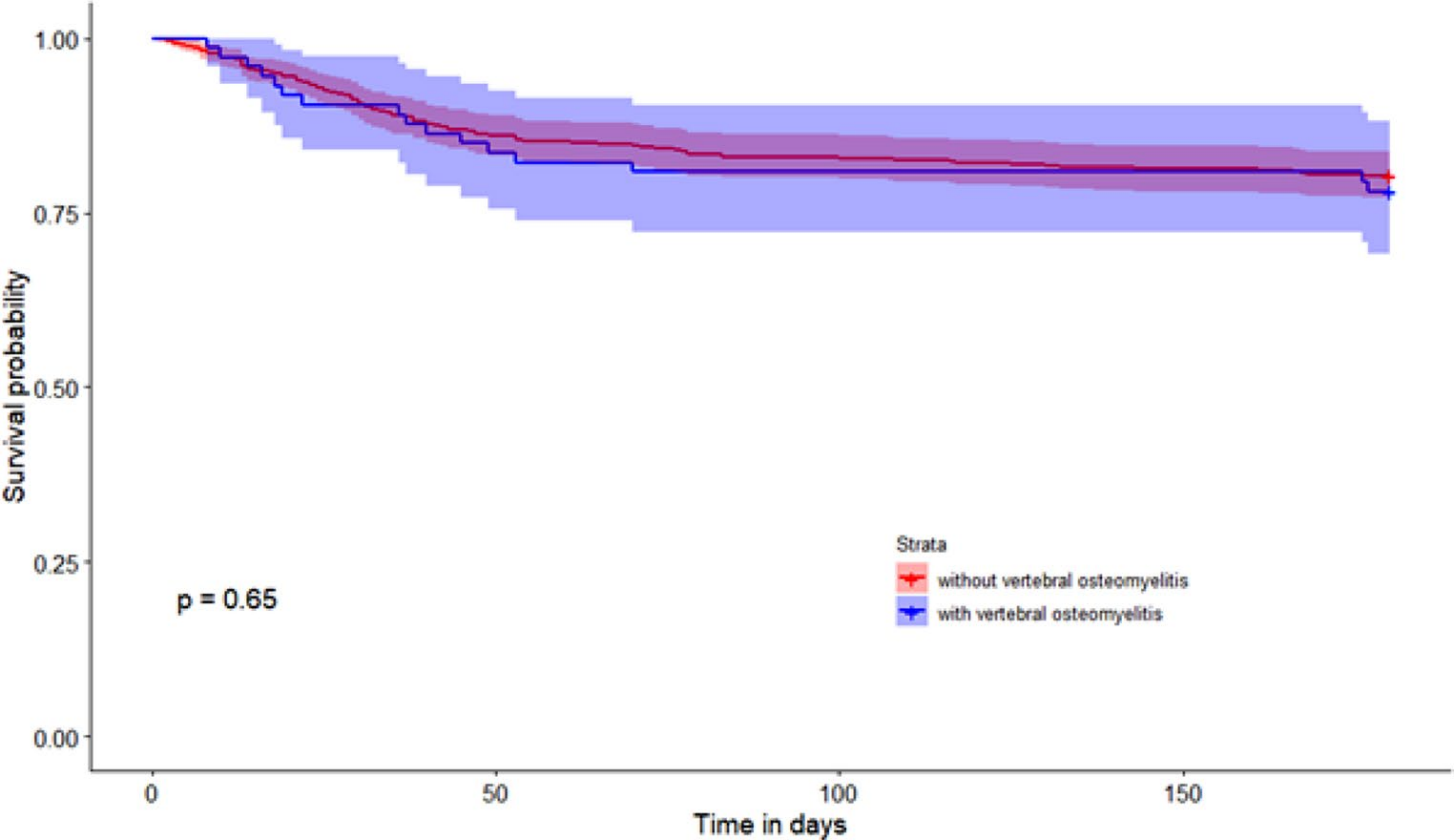
6 month mortality in patients with infective endocarditis with and without VO

At 6 months after diagnosis, 126 patients (19.9%) had died: 16/73 (21.9%) patients with VO versus 110/560 (19.6%) patients without VO (*p* = 0.65). No difference in the 6-month mortality rate between the groups was found in the Kaplan-Meier analysis(Fig. 1). The mortality was not affected by whether the patient was initially diagnosed with IE or VO (9/36 vs. 7/37; p: 0.82). No significant difference between the two groups was found in proportion of patients undergoing cardiac surgery or device extraction (Table 3.).

**Table 3:** Clinical outcome of patients with infective endocarditis with or without vertebral osteomyelitis

	With vertebral osteomyelitis (*n* = 73)	Without vertebral osteomyelitis (*n* = 560)	OR(95% CI)
**Clinical outcomes**			
Cardiac surgery	3 (4.1)	31 (5.5)	0.95 (0.35–2.15)
Device extraction	4 (5.5)	27 (4.8)	1.14 (0.30–3.66)
6-month all-cause mortality	16 (21.9)	110 (19.6)	1.13 (0.67–1.91)

Data are reported as number of patients (%)

Information about relapse data was available for patients treated in the NWZ hospital (*n* = 165). A total of 9.1% (15/165) of patients experienced a relapse. No statistically significant difference was found in the odds of having relapse of infective endocarditis 6 months after diagnosis in patients with and without VO (5/31, 16.1% vs. 10/134,7.5%, OR 3.62, 95% CI 0.94–13.34).

## Discussion

In this multicenter prospective study of 633 patients with infective endocarditis, the rate of concomitant VO was 11.5%. In 51% of IE cases co-occurring with VO, the diagnosis of VO preceded the diagnosis of IE. We found age and *Enterococcus spp*. to be associated with a higher risk for concomitant VO. This association remained in patients with definite IE and patients without VO at onset. Finally, we found no significant difference in crude 6-month mortality or relapse rates in IE patients with and without VO.

Vertebral osteomyelitis is a serious condition which can lead to long term complications, including spinal instability and neural compression. In this study we found a VO prevalence of 11.5%, and complications of VO were not infrequent (36%). This is slightly higher in comparison with recent studies that reported an prevalence of 8–10% [2, 3]. One explanation for this difference could be the high rate (59.1%) of ^18^F-FDG-PET/CT performance in our study. When comparing patients with and without ^18^F-FDG-PET/CT scan, VO was more frequently diagnosed in the PET-CT group (15.5% vs. 5.8%, *p* < 0.001).

An important observation was that among patients with VO and IE, the diagnosis of VO was established before IE in 51% of cases. While recent guidelines on treatment and diagnosis of IE recommend searching for IE in all patients with pyogenic VO and positive blood cultures [12], VO is however not routinely screened for in patients with IE.

In our study, the majority of patients with VO experienced back pain (77%), emphasizing its potential utility as a symptom warranting further investigation. However, in 23% of cases VO was asymptomatic and VO might therefore go undetected if it is not actively screened for. Identifying risk factors for VO is important for guiding additional diagnostic studies in at-risk patients. Prompt diagnosis of VO can subsequently influence the duration of antimicrobial therapy and inform other interventions, such as drainage.

We found age to be associated with a higher risk of VO. This may stem from the changing vascular anatomy of the vertebrae during life, resulting in more sludging of the blood flow in the metaphysis of the vertebrae [13, 14]. This could predispose to hematogenous bacterial seeding in the vertebrae, explaining the higher incidence of VO in the older patient population [6, 15]. This is similar to a previous study, which also found age to be associated with VO [2].

Enterococci are increasingly important pathogens in infective endocarditis, due to the ageing population and an increasing number of invasive medical procedures [16]. Enterococci are consistently reported as a risk factor associated with VO in patients with IE [2, 3]. This is in concordance with previous findings which found that in patients with enterococcal bacteremic osteoarticular infections, 91% (10/11) also had IE. Enterococci possess various virulence factors, which include adhesion proteins and biofilm formation capabilities. These factors facilitate adherence, invasiveness and the ability to cause infections, particularly in the urinary tract, and these factors may potentially also contribute to infections of the vertebrae [17, 18]. Previous studies also described the association between streptococcal IE (*S. gallolyticus*,* S. viridans*) and VO [2, 3]. We could not reproduce these findings.

Although no statically difference was observed in mortality and relapse rates between patients with and without VO, relapses occurred numerically more frequently in patients with concomitant VO (OR 3.62, 95% CI 0.94–13.34). Del Pace et al. [3] reported similar findings in patients with VO and IE, noting a significantly higher incidence of relapses among patients with concurrent VO compared to those without VO (17.2% vs. 4.2%, *p* < 0.01). However, in multivariable analysis only a history of drug abuse remained as an independent predictor of IE relapses (*p* = 0.009, HR 6.8 CI 1.6–29). In our study information regarding drug abuse was unavailable.

New compared to previous research is that we also investigated risk factors after exclusion of those patients who were already diagnosed with VO at the moment of IE diagnosis, in order to identify predictors for VO in IE patients not yet diagnosed with VO. Additionally, this is the largest study to date to include prospective data of the past decade in both a university and general teaching hospital [2].

Our main limitation is that imaging was not protocolized and it is likely that imaging has been performed less often in patients without perceived symptoms of VO, which could lead to detection bias and therefore to underdiagnosis of VO. On the other hand, it is plausible that imaging was performed more frequently in specific patient groups, e.g. patients with a prosthetic heart valve. Of note, a majority of participants underwent PET-CT scan (58%), a modality with a high sensitivity of detecting VO lesions [19]. Second, in this study we did not correct for survival bias, i.e. the patient had to survive until imaging for the VO diagnosis to be established. This may have led to an underestimation of the prevalence of VO [20]. 

The 2023 update of the ESC guidelines marks a significant shift in the diagnostic criteria for endocarditis, now including *Enterococcus faecalis* and hematogenous osteoarticular complications, such as spondylodiscitis, as major and minor criteria, respectively. This revision reflects the growing recognition of the potential impact of these factors on the diagnosis of endocarditis. If the 2023 criteria were applied to this cohort, we anticipate that a greater number of patients would be diagnosed with Duke definite endocarditis [12]. The timing of surgery in relation to the diagnosis and treatment of endocarditis was not considered, which could influence the interpretation of outcomes. Early surgical intervention is known to have a significant impact on prognosis [21, 22]. Finally, in only a subset of patients information about relapse was collected, most likely resulting in a underestimation of the relapse risk in both groups. Given the small number of patients who experienced a relapse, it was not possible to identify risk factors associated with relapse in this cohort.

In conclusion, the prevalence of VO in IE patients is 11.5%. Higher age and infection caused by enterococci are associated with VO. Among older patients and patients with IE caused by enterococci, there should be a higher index of suspicion for vertebral infection.

## Data Availability

No datasets were generated or analysed during the current study.
